# Technical Quality of Root Canal Treatment Performed By Undergraduate Dental Students

**Published:** 2008-07-10

**Authors:** Bahareh Dadresanfar, Nahid Mohammadzadeh Akhlaghi, Mehdi Vatanpour, Hojat Atef Yekta, Ladan Baradaran Mohajeri

**Affiliations:** 1 *Department of Endodontics, Dental School, Islamic Azad University/ Iranian Center for Endodontic Research, Tehran, Iran*; 2 *General Practitioner, Tehran, Iran*

**Keywords:** Dental Student, Endodontics, Errors, Iatrogenic, Quality, Radiograph

## Abstract

**INTRODUCTION:** This study was carried out to evaluate the technical quality of root canal treatment (RCT) performed by undergraduate dental students at the Islamic Azad University in Tehran, Iran.

**MATERIALS AND METHODS:** Four-hundred records of patients who had received RCT at faculty of dentistry, between the years 2004-2006 were evaluated. For each treated tooth at least three periapical x-rays were assessed: preoperative, working length measurement, and postoperative. Evaluation of root canal filling was based on two variables: length and density. The filling length was recorded as adequate, under- or overfilled. Density of filling was recorded as poor or adequate. Fillings with adequate length and density were recorded as acceptable. Detected iatrogenic errors were: ledge formations, root perforations, furcation perforations, strip perforations and presence of fractured instruments. Results were evaluated statistically using one-way ANOVA and Chi-square analysis.

**RESULTS:** Out of the 400 teeth, 50.5% had at least one of the mentioned errors. Acceptable filling was observed in 32.5% of all studied teeth. Ledge was found in 17.5% of the teeth. Canal curvature was the most important factor associated with ledge formation (P<0.05).

**CONCLUSION:** The technical quality of RCT performed by undergraduate dental students using step-back preparation and cold lateral condensation was classified as acceptable in 32.5% of the cases.

## INTRODUCTION

Endodontic treatment is an essential part of comprehensive quality care. Controlled studies have shown that RCT brings high success rates of more than 90% (1,2). Success or prognosis of RCT has been shown to depend on many variables one of which is the technical quality of the root canal filling (3,4).

Smith *et al. *(5) reported that the canal preparation technique and the root canal filling length, relative to the radiographic apex, significantly affect the success of conventional RCT. In most studies it is mentioned that periradicular health is associated to adequate density root filling that terminate within radiographic apical 2 mm (6-9). It is also of particular importance to outline that the sequence of interdependent steps characteristic of RCT may be interrupted or even fail at any time or stage of the process due to iatrogenic complications. Procedural errors compromise root canal cleaning and shaping, and result in incomplete root filling and jeopardize the outcome of the treatment (10). The study of the treatment quality and prevalence of different procedural errors can help improving educational programs and lead the society to a higher level of health services.

The purpose of this study was to evaluate the technical quality of root fillings and to identify the presence of ledges, root perforations, furcation perforations and fractured instruments, in cases treated by undergraduate students at Islamic Azad University.

**Table 1:** The criteria followed to record root filling quality based on x-rays

Variable	Criteria	Definition
Lenght of root canal filling	Adequate	Root filling ending ≤ 2 mm short of radiographic apex
	Over	Root filling ending beyond the radiographic apex
	Under	Root filling ending ≥ 2 mm short of radiographic apex
Density of root canal filling	Poor	Not uniform density of root filling with clear presence of voids and canal space
	Adequate	uniform density of root filling without visible voids and canal space

**Table 2:** Number (%) of poor root fillings in molars

	Number	Ledged teeth	Percentages
**Maxillary 1**^st^** molar**	53	32	60.4
**Maxillary 2**^nd^** molar**	10	2	20
**Mandibular 1**^st^** molar**	82	35	42.7
**Mandibular 2**^nd^** molar**	34	10	29.4

## MATERIALS AND METHODS

For this retrospective study we investigated random samples of 640 records belonging to patients who had received RCT at Faculty of Dentistry, Islamic Azad University, Tehran, Iran during 2004-2006. Records of patients younger than 16 years of age or older than 68 years of age were excluded. Records without pre- and postoperative periapical x-rays, good radiographic quality, complete RCT, and retreated cases were excluded. The final samples consisted of 400 teeth with obturated root canals.

All RCTs were carried out by undergraduate students using step-back technique with hand instrumentation and lateral compaction filling technique using gutta-percha (AriaDent, Tehran, Iran) and AH26 sealer (Dentsply, DeTrey, Konstanz, Germany). For each tooth, at least three radiographic images were examined: preoperative, working length determination, and postoperative. Two investigators utilizing a magnifying lens examined the x-rays, independently. The results were compared and a final consensus was agreed on. In cases of disagreement, a third investigator was asked to read the x-ray for final agreement. The technical quality of the root canal filling and the presence of iatrogenic errors depicted in x-rays were evaluated and classified. The recorded data included root canal type, curvature, periapical pathosis, length and density of the filling, presence of a ledge, root perforation, strip perforation, furcation perforation and fractured instrument in each examined root canal. In cases of maxillary and mandibular multi-canalled teeth, which were imaged with altered horizontal angulation, it was assumed that they had been imaged with mesial angulation. As a result, according to the buccal object rule, it was possible to differentiate the lingual from the buccal root canal in multi-rooted teeth. The criteria for radiographic classification were as follows:

- Ledge formation was diagnosed when the root filling was at least 1 mm shorter than the initial working length and deviated from the original canal path in teeth with root canal curvature.

- Furcation perforation was diagnosed when extrusion of filling material through the furcation area was detected in multi-rooted teeth.

- Strip perforation was diagnosed when extrusion of filling material was detected in the outer wall of mesiobuccal roots of maxillary molars, mesial roots of mandibular molars and in any root of other teeth.

- Presence of a fractured instrument was diagnosed when one was detected inside a root canal or extending into the periapical area.

- Presence of radiographic pathosis was recorded when the diameter of the radiolucency exceeded twice the width of the lateral periodontal ligament space.

- Canal curvature was assessed according to Schneider (11) and if canal curvature was <30**o**, it was recorded as straight and if canal curvature was >30**o**, it was recorded as curved.

- The length and density of root canal filling were recorded according to Table 1.

Statistical analysis of the data was performed using SPSS 13.0 for windows. One-way ANOVA was used to determine statistically significant differences between the quality of RCTs and the iatrogenic accidents occurring due to the curvature of the teeth, and chi-square analysis to determine statistically significant differences between the technical quality and also iatrogenic accidents to the tooth type. The significance level was set at P<0.05.

**Table 3.** The frequency (percent) of mishaps in different teeth

	N (%)	Ledge	Strip preforation	Cervical preforation	Furcal preforation	Poor filling	Under filling	Over filling	Broken
Maxilary	63	0	0	1	0	11	2	9	0
Incisor	(74.1)	(0)	(0)	(1.6)	(0)	(17.5)	(3.2)	(14.3)	(0)
Mandibular	22	0	1	0	0	4	0	3	0
Incisor	(25.9)	(0)	(4.5)	(0)	(0)	(18.2)	(0)	(13.6)	(0)
Maxilary	78	6	0	0	0	15	14	11	1
Premolar	(57.4)	(7.7)	(0)	(0)	(0)	(19.2)	(17.9)	(14.1)	(1.3)
Mandibular	58	6	1	0	0	8	6	3	0
Premolar	(42.6)	(10.3)	(1.7)	(0)	(0)	(13.8)	(10.3)	(5.2)	(0)
Maxilary	63	18	7	0	0	34	15	23	1
Molar	(35.2)	(28.6)	(11.1)	(0)	(0)	(54)	(23.8)	(36.5)	(1.6)
Mandibular	116	40	7	0	1	45	38	29	2
Molar	(64.8)	(34.5)	(6)	(0)	(0.9)	(38.8)	(32.8)	(25)	(1.7)

## RESULTS

***General findings***

Six hundred-forty records were investigated. The records that did not include pre/postoperative x- rays (8.3%), those with previous RCTs (33.4%) and those with poor radiographic quality (58.3%) were excluded (n=240). Four hundred teeth (836 canals consisting of 44.75% molars, 34% premolars and 21.25% anterior teeth) were included in this study. Of all teeth, 50.5% had at least one of the studied errors, 49.5% of all teeth were error free, and 74% of the teeth had curved canals. Also 58.1% of curved canals had at least one of the errors, 37.5% of the teeth had periapical pathosis and 61.3% of these teeth had at least one of the errors studied in this research.

***Iatrogenic errors***

Ledge formation was found in 17.5% of the teeth. A significant difference in the frequency of ledged root canals was found between anterior teeth (0%), premolars (18%), and molars (63.1%) (P<0.05). The incidence of ledged canals was significantly higher in mandibular teeth than maxillary teeth (P<0.05). The first mandibular molar exhibited the highest incidence of ledged root canals in all the teeth.

Ledging was found in 3.2% of straight and in 19.1% of curved canals (P<0.05). Canal curvature was the most significant factor affecting the incidence of ledges in all teeth (P<0.05).

Strip perforation was detected in 16 of the 400 teeth (4%). There was no significant relation between the incidence of strip perforation and the canal curvature or any of the other factors studied here.

There was one cervical and one furcation perforation. Fractured instruments were present in 4 root canals.

Poor root fillings were detected in 29.25% of all the treated teeth. The majority were observed in curved canals. Of the curved canals, 29% had poor fillings as opposed to 12.6% of straight root canals. The relationship between density of the root canal filling and the presence of curvature was statistically significant (P<0.05 and P<0.05). There was a significant difference between the percentages of poor root canal fillings in molars and other teeth (P<0.05). According to table 2, the first maxillary molar exhibited the highest rate (60.4%) of poor root fillings.

Table 3 shows the frequency of mishaps in different teeth. It should be mentioned that some of the procedural accidents in this table have overlaps. Over filling and under-fillings were found in 19.5% and 18.5% of the root canals, respectively. The relationship between overfilling and the presence of periapical pathosis was statistically significant (P<0.05).

Adequate filling length and the absence of voids in obturation were prerequisites for acceptable root fillings (32.5%).

## DISCUSSION

Periapical radiographs of patients who received RCT were used as the main source of information for assessing the prognosis. All the radiographs used were taken during routine RCT procedures within a dental student practice and were not taken for this study. More than half of all the periapical x-rays were classified as unreadable as a result of poor radiographic technique or processing. It should also be said that the radiographs taken were not standardized; inevitable changes in beam and film angulation between pre- and postoperative views affected the images of teeth and bone. Therefore, the radiographs were examined by three operators; other studies have suggested this method for evaluation as well (12,13).

The radiographic criteria used to assess the quality of RCT were the same as those used in previous studies (12-14). The percentage of root fillings with adequate length was 62% in the present study. Although it is difficult to compare these results with other studies, the percentage of root fillings that had adequate length was greater when compared with those reported by Lupi-Pegurler *et al. *(38.7%) (15) and Boltacz- Rzepkowska and Pawlicka (48.9%) (14); our results were almost in the same range as those reported by Chueh *et al. *(16). However estimation of the root filling length was probably not reproduced correctly in all radiographs because postoperative radiographs taken by undergraduate students using bisecting-angle technique. Forsberg demonstrated that root fillings are projected shorter, ie, more coronally on the x-rays exposed with the bisecting-angle technique than with the paralleling technique (17). The 18.5% under filling in this study is in accordance with Er *et al. *results (13); as Barrieshi-Nusair *et al. *report (12) mandibular molars have higher percentage of short fillings. This may be explained by the anatomy of these teeth ie, multi-canalled roots and their curvature, making root canal treatment more challenging for the students.

Although Barrieshi-Nusair *et al. *(12) and Peak *et al. *(3) reported more than 30% under filling in their studies; these differences might be due to the fact that in our study only one technique of root canal therapy was carried out by students, while for example several techniques were performed by different practitioners in another study (3).

In this study over filling was found in (19.5%) of all the teeth. Periapical lesion was found to be the most significant factor affecting the incidence of over filling. Peak *et al. *(3) reported 18% of over fillings in their study and found that in the presence of pre-existing periapical radiolucency, the over filling seemed to be of some benefit for the success rate. The lower percentage of over fillings reported by other studies (10,12,18) may be due to the fact that 37.5% of the teeth in the present study had pre- existing periapical radiolucency. Periapical lesions can result in resorption and destruction of the apical constriction and this loss may have influenced working length control by undergraduate students.

Poor root fillings occurred in 29.3% of the teeth. While this concurs with Barrieshi- Nusair *et al. *(12) who reported 27.4% poor density fillings, it varies with other studies (13,19). In curved canals the prevalence of poor root filling was higher (29%) than straight canals (12.6%). Although Barrieshi-Nusair *et al. *(12) reported no difference in the density of root fillings among the different tooth groups, in the present study the highest percentage of poor filling was found in maxillary first molars (60.4%). Another study has reported that maxillary molars had the highest incidence of poor root filling (52.1%) (13). Obviously, difficult access to posterior teeth, multi-canalled anatomy and curved roots are responsible for these challenges.

The frequency of root canals with "acceptable filling" was 32.5%. This finding is in complete accordance to the 33% reported by Er *et* *al.* (13) and the 31% reported by Hayes et al. (18). However, higher percentages of acceptable filling have been reported (10,12). These differences could be due to many reasons, which may include factors such as the design of the study, the criteria followed and the techniques used for root canal treatment.

This study considered a ledge present when the root filling was at least 1mm shorter than the working length and deviated from the original canal curvature. A short obturation may also be due to packed dentinal chips or residual debris forced apically during instrumentation, resulting in apical canal blockage.

It has been shown that step-back technique can induce such clinical complications and consequently may have contributed to an increased ledge incidence rate. The results indicated that 17.5% of all the canals had been ledged. Anterior teeth and pre-molars were ledged less frequently than molars. This probably occurred as a result of the prevalence of narrow and curved canals in molars. Preparation of curved canals is challenging due to the problem of the canal straightening by the inherent restoring forces of the file design and alloy.

In molars, a ledge was present in 63.1% of the cases. This is almost in a same range with 51.5% reported by Kapalas and Lambrianidis (20). Also curved canals have higher prevalence of ledge formation (19.5% versus 3.2%).

Using large files without adequate attention to anti-curvature filing technique by un-skilled undergraduate students might explain the presence of strip perforations in this study. The low incidence of other iatrogenic errors in this study may have had a negative effect on detecting statistically significant differences.

Important adjuncts in modern endodontic treatment such as nickel-titanium rotary instruments and electronic apex locators were not used in this study. Plans are being made to incorporate usage of electronic apex locators and nickel titanium rotary systems into routine preclinical and clinical educational courses.

## CONCLUSION

The technical quality of root canal treatment performed by undergraduate dental students using step-back preparation and cold lateral condensation was classified as acceptable in 32.5% of the cases. Canal curvature was the most important clinical factor affecting the results of this study.
